# Integrated genomic characterization of cancer genes in glioma

**DOI:** 10.1186/s12935-017-0458-y

**Published:** 2017-10-13

**Authors:** Aijun Liang, Bin Zhou, Wei Sun

**Affiliations:** Department of Neurosurgery, Jiangxi Provincial People’s Hospital, No. 92, Aiguo Road, Nanchang, 330006 Jiangxi Province China

**Keywords:** Glioma, Driver gene, Driver pathway

## Abstract

**Background:**

Cancers are caused by the acquisition of somatic mutations. Numerous efforts have been made to characterize the key driver genes and pathways in glioma, however, the etiology of glioma is still not completely known. This study was implemented to characterize driver genes in glioma independently of somatic mutation frequencies.

**Methods:**

Driver genes and pathways were predicted by OncodriveCLUST, OncodriveFM, Icages, Drgap and Dendrix in glioma using 31,958 somatic mutations from TCGA, followed by an integrative characterization of driver genes.

**Results:**

Overall, 685 driver genes and 215 driver pathways were determined by the five tools. *FSTL5*, *HCN1*, *TMEM132D*, *TRHDE* and *KRT222* showed the strongest expression correlation with other genes in the co-expression network of glioma tissues. *ST6GAL2*, *PIK3CA*, *PIK3R1*, *TP53* and *EGFR* are at the core of the protein–protein interaction network. 133 driver genes were up-regulated and associated to poor prognosis, 43 driver genes were down-regulated and related to favorable clinical outcome in glioma patients. The driver genes such as *MSH6* and *RUNX1T1* might serve as candidate prognostic biomarkers and therapeutic targets in glioma.

**Conclusions:**

The set of new cancer genes and pathways sheds insights into the tumorigenesis of glioma and paves the way for developing driver gene-targeted therapy and prognostic biomarkers in glioma.

**Electronic supplementary material:**

The online version of this article (doi:10.1186/s12935-017-0458-y) contains supplementary material, which is available to authorized users.

## Introduction

Gliomas are tumors that arise from glial cells and common primary brain tumors in adults, with an incidence rate of 6.03 per 100,000 in USA [1]. Gliomas are classified into a variety of subtypes, including astrocytoma, glioblastoma, oligodendroglioma, ependymoma, mixed glioma, malignant glioma, and a few more rare histologies. Of them, astrocytoma accounts for about 70% of glioma cases [1]. Low grade glioma is a lethal disease in young adults, with an average survival time of 7 years, only 20% of low grade glioma patients survived for more than two decades [2].

Recently, large-scale genomics studies have been conducted to determine the core genes and pathways underlying gliomagenesis and to define molecular subtypes in glioblastoma and lower-grade gliomas [3–5]. For instance, 87% of glioblastomas have genetic alterations in the *TP53*/*MDM2/MDM4/p14ARF* pathway, including *TP53* mutations or homozygous deletion (35%), *MDM2* amplification (14%), *MDM4* amplification (7%), or *p14ARF* homozygous deletion or mutation (49%) [3]. *IDH1* mutation combined with either *TP53* mutation or total 1p/19q loss is a frequent and early change in the majority of common adult gliomas but not in primary glioblastomas [6]. Lower-grade glioma patients with an IDH mutation and 1p/19q co-deletion showed favorable clinical outcomes in lower-grade gliomas [5]. The DNA repair enzyme *MGMT* is frequently methylated in glioma, methylation of the CpG islands of the *MGMT* gene prevents transcription, which may increase the sensitivity of glioma to alkylating agents [7–9].

Though considerable progresses have been achieved, the etiology of glioma is still not completely understood. In this study, we performed genome-wide analyses of 576 gliomas, incorporating exome sequence, mRNA expression, protein–protein interaction, DNA copy number variations and clinical outcome from The Cancer Genome Atlas (TCGA) database. We revealed a list of cancer-driving genes and pathways and many driver genes were aberrantly expressed, co-expressed with other driver genes, involved in copy number variations and correlated with prognosis of glioma patients. Our study is of importance to characterize cancer biology and identify potential therapeutic targets and prognostic biomarkers in glioma.

## Methods and materials

### Classification of cancer mutations

31,958 somatic mutations generated by whole-exome sequencing of 576 pairs of glioma tumor/normal samples were downloaded from TCGA database at Broad Institute (http://firebrowse.org/?cohort=GBMLGG&download_dialog=true, download on April 15, 2017) [10]. Functional impact of somatic mutations was evaluated by Ensembl Variant Effect Predictor (VEP) [11] and then all mutations were classified into 11 categories according to their functional impact.

### Prediction of driver genes and pathways

Driver gene candidates were predicted by five distinct tools, including OncodriveCLUST [12], OncodriveFM [13] (https://www.intogen.org), drgap [14] (https://code.google.com/archive/p/drgap), icages [15] (http://icages.wglab.org/) and Dendrix [16] (http://compbio.cs.brown.edu/projects/dendrix/) with default parameters. The following criteria were applied to determine driver genes or pathways: (1) genes have q values smaller than 0.05 (OncodriveCLUST and OncodriveFM), (2) genes or pathways have adjusted P values less than 0.05 (drgap), (3) genes were classified as drivers by icages and showed icagesGeneScores above 0.5 (icages), (4) the selected genes were sampled at least 10% (100/1000) of the times in the mutually exclusive analysis (Dendrix). Then, 576 glioma patients were stratified into *IDH1*-mutated (236) and non-*IDH1*-mutated (340) groups. Driver gene and pathway analyses were performed in the two groups using the same methods as described above.

### GO, KEGG pathway enrichment analyses

In order to characterize the functional enrichment of driver genes, GO term and KEGG pathway enrichment analyses were implemented for all the driver genes on the home page of STRING [17] (http://string.embl.de/). GO terms and KEGG pathways were considered to be significantly enriched for driver genes with the cutoff of false discovery rate (FDR) < 0.05.

### Expression and co-expression network analyses

RNA-seq data of 75 glioma and 17 normal brain tissues were obtained from the study of Gill et al. (GSE59612) [18]. Gene expression values expressed as Fragments per kilobase of exon per million fragments mapped (FPKM) were compared between glioma and normal brain tissues for all driver genes with t test, P values were adjusted using False Discovery Rate (FDR) in R. Genes were regarded as significantly differentially expressed with the cutoff of adjusted P value < 0.05. Next, Co-expression network was constructed by weighted correlation network analysis (WGCNA) using gene expression log2(FPKM + 1) [19]. All parameters were used with the default values except for the softpower (12) and threshold (0.004). Degree centrality is defined as the number of connections one node has to another. Hub genes which have the highest degrees connect most adjacent genes and build the structure of the network.

### Protein–protein interaction network analysis

Protein–protein interaction (PPI) network was constructed using STRING to prioritize the core driver genes in glioma. As for each driver gene, combined STRING scores of all protein–protein interactions were summed as total STRING score which represents the number of interactions the driver gene has with other genes.

### Sources of copy number variation and survival analyses

Focal copy number variations (CNVs) were acquired from 52 glioma samples at broad institute [10] (http://firebrowse.org/?cohort=GBMLGG&download_dialog=true#). TCGA RNAseq and clinical outcome data were retrieved to assess whether the expression of driver genes could be associated to patients’ survival in glioma. Multivariate Cox regressions were performed with the coxph function from the R survival library using gene expression, sex, age, and grade or histology as multivariates [20]. Kaplan–Meier survival curves were plotted on the website of oncolnc [21] (http://www.oncolnc.org/), log rank test was used to compare the survival rates between high and low expression groups which refer to 25% (127/508) of glioma patients that have the highest or lowest RNA expression levels respectively.

## Results

### The catalogue of somatic mutations

**Fig. 1:**
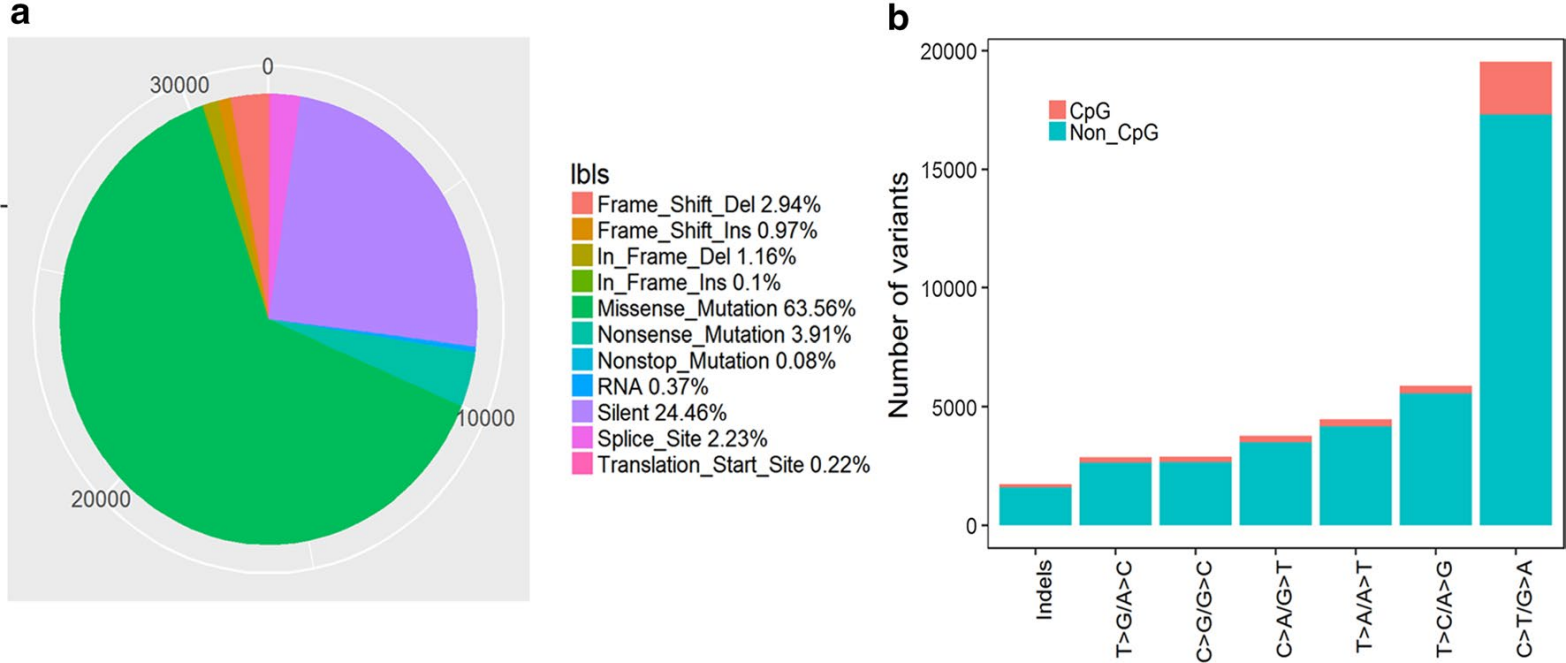
Characterization of somatic mutations in glioma. **a** The number and proportion of mutation classes with different functional impact in glioma. **b** The somatic mutation signatures in glioma

31,958 somatic mutations comprise 30,216 single-nucleotide variants (SNVs) and 1742 small insertions or deletions (indels). The SNVs included 7818 silent, 20,311 missense, 1248 nonsense, 713 splice-site, 119 RNA, 71 translation start site and 24 nonstop mutations, 1259 indels caused reading frame shifts, 404 indels were in frame mutations. Over 67.46% (21,559/31,958) of variants were non-synonymous mutations (Fig. 1a). C>T/G>A, T>C/A>G and T>A/A>T accounted for 54.18, 17.38 and 13.05% of the variant types in the non-CpG sites, 6.90, 1.06 and 0.98% of variant types in the CpG islands respectively. Therefore, C>T/G>A, T>C/A>G and T>A/A>T were the three predominant transitions in glioma (Fig. 1b).

### Cancer driver genes and pathways in glioma

**Fig. 2:**
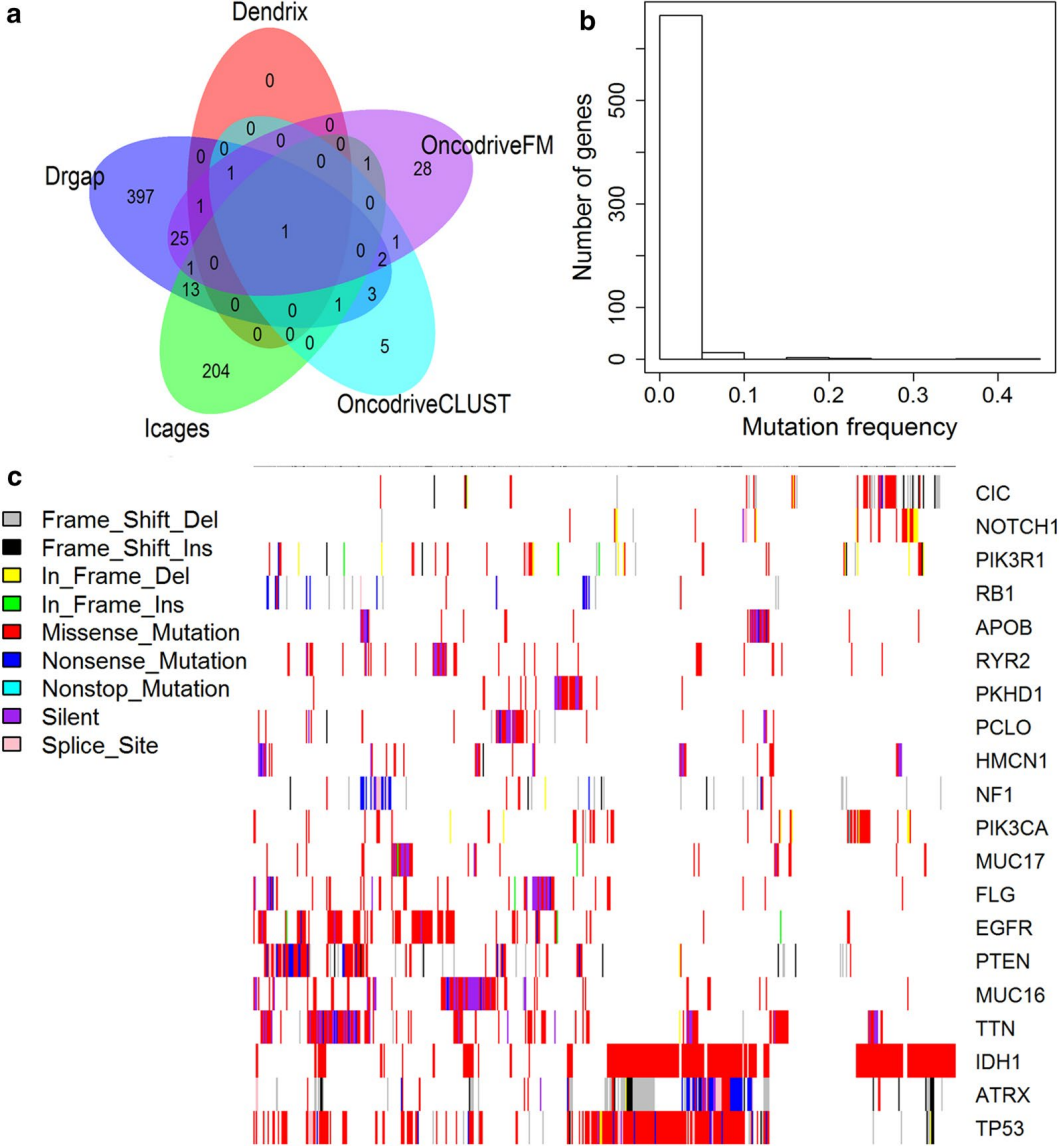
Driver genes in glioma. **a** The overlap of driver genes predicted by five distinct methods. **b** The distribution of mutation frequencies of driver genes. **c** The mutation patterns of top 20 most frequently mutated driver genes across 576 glioma samples

Overall, there were 15, 68, 221 and 445 driver genes determined by OncodriveCLUST, OncodriveFM, icages and drgap respectively (Additional file 1: Table S1). Dendrix reported 11,814 genes mutated in at least one patient. We performed Dendrix analyses for sets of size ranging from 2 to 4. When k = 2, the pair *IDH1* and *PTEN* was sampled 95.3% (953/1000) of the times. When k ≥ 3, the triple *IDH1*, Unknown and *PTEN*, *IDH1*, *EGFR* and *PTEN* were sampled 69.2% (692/1000) and 30.7% (307/1000) of the times respectively. Therefore, *IDH1*, *PTEN* and *EGFR* showed mutational exclusivity in glioma (Additional file 1: Table S1). *EGFR* is the only common gene detected by all five tools (Fig. 2a), suggesting that *EGFR* plays a pivotal role in the tumorigenesis of glioma. Of 685 driver genes, the majority of driver genes are low-frequency mutated genes in glioma, with an average mutation rate of 1.39% (Fig. 2b). *IDH1*, *TP53*, *ATRX*, *TTN*, *PTEN*, *EGFR* and *MUC16* were known recurrently mutated genes and showed mutation rates of 40.97, 39.93, 23.78, 22.92, 17.88,16.32 and 15.10% across all samples (Fig. 2c). Besides the list of driver genes, Drgap also reported 215 driver pathways in glioma, such as Hedgehog signaling pathway, *mTOR* signaling pathway, glioma and acute myeloid leukemia (Additional file 1: Table S2).

The *IDH1* gene encoding isocitrate dehydrogenase 1 (*IDH1*) catalyzes the oxidative carboxylation of isocitrate to a-ketoglutarate, causing production of *NADPH* in the citric acid (Krebs) cycle [22]. *IDH1* is frequently mutated in the majority of low grade gliomas and secondary high grade gliomas. *IDH1* mutations change the function of the enzymes, increase DNA methylation and correlate with improved prognosis in glioma [23]. 576 glioma patients were classified into *IDH1*-mutated (236 patients) and non-*IDH1*-mutated groups (340 patients) based on the occurrence of *IDH1* mutation. Then we applied OncodriveCLUST, OncodriveFM, icages, drgap and Dendrix to detect driver genes and pathways in the two distinct groups. The number of driver genes detected by the five tools was largely different between *IDH1*-mutated and non-*IDH1*-mutated groups. There were only 2, 5, 46, 8 and 1 overlapping driver genes predicted by OncodriveCLUST, OncodriveFM, icages, drgap and Dendrix respectively (Additional file 1: Figure S1, Tables S3, S4). Moreover, a set of driver genes were predicted to be *IDH1*-associated, such as *IDH1, NOTCH1, FUBP1, ARID1A, KAT6B* and *ARID2*, while others were *IDH1*-independent, such as *EGFR, PIK3CA, BRAF, RB1* and *PTGS2* (Additional file 1: Tables S3, S4, Figure S1). However, the majority of driver pathways (196/209, 93.78%) unraveled by drgap in glioma patients with *IDH1* mutations overlapped with those (196/214, 91.59%) in glioma patients without *IDH1* mutations. All the results suggest that *IDH1* and non-*IDH1* mutated glioma subtypes are caused by mutational disruption of different genes but similar pathways.

### GO term and KEGG pathway enrichment analyses

The enrichment of GO terms and KEGG pathways was analyzed for 685 driver genes on the home page of STRING. GO enrichment analysis indicated driver genes were significantly enriched in 1750 biological processes (FDR < 0.05). The main GO biological process terms showed a wide spectrum of functional processes ranging from cellular developmental process, cell differentiation, programmed cell death, apoptotic process to regulation of metabolic processes (Additional file 1: Table S5). STRING also revealed 145 KEGG pathways significantly enriched for driver genes, such as glioma, melanoma, thyroid cancer, pancreatic cancer, renal cell carcinoma, bladder cancer, colorectal cancer, non-small cell lung cancer, endometrial cancer, prostate cancer, acute myeloid leukemia, *MAPK* signaling pathway, *mTOR* signaling pathway and cell cycle (FDR < 0.05, Additional file 1: Table S6).

### Expression profiling in glioma

**Fig. 3:**
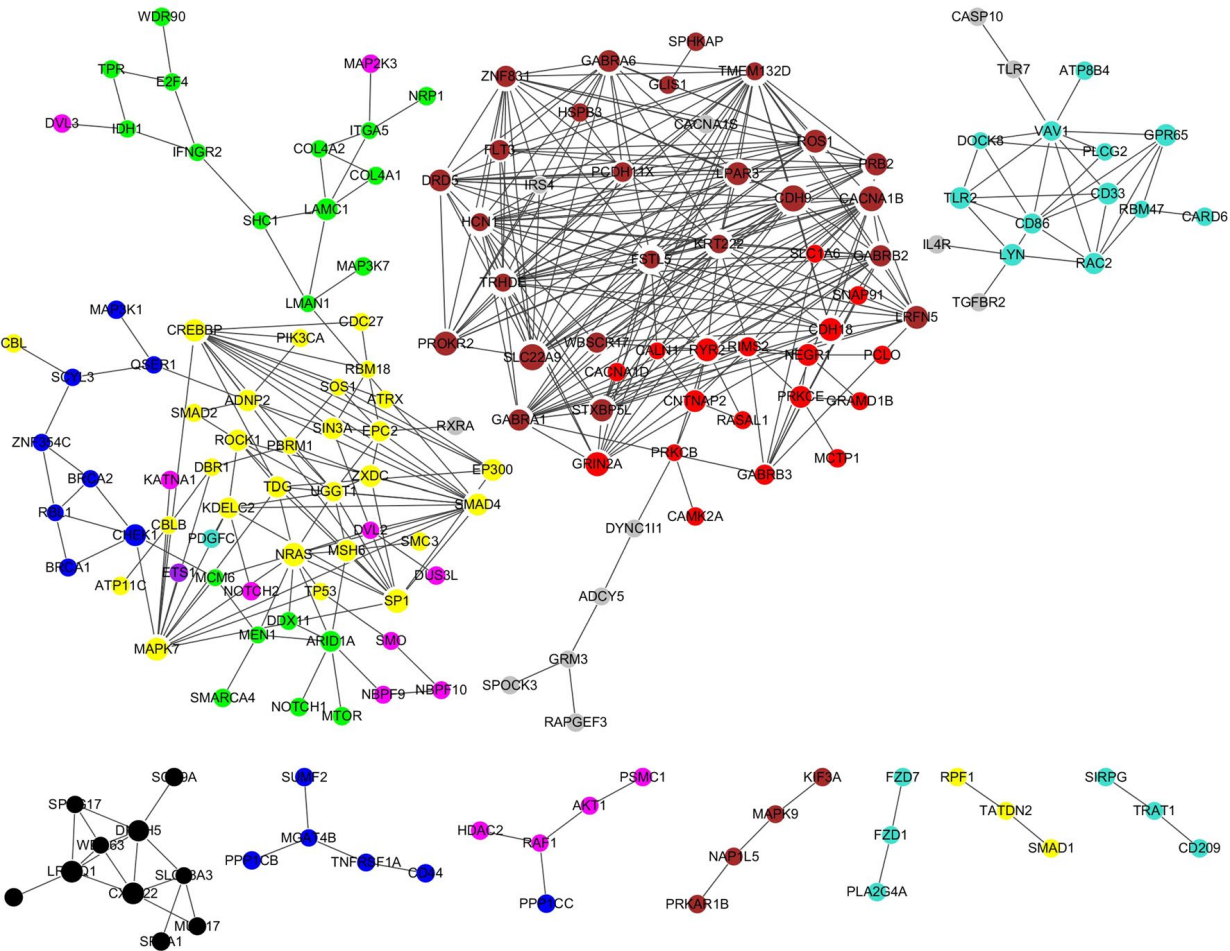
Co-expressed differentially expressed driver genes and their network in glioma tissues; Of note, the greater the sizes of nodes are, the stronger the differentially expressed driver genes are co-expressed with surrounding genes. The nodes that have the same color belong to the same module detected by WGCNA

**Fig. 4:**
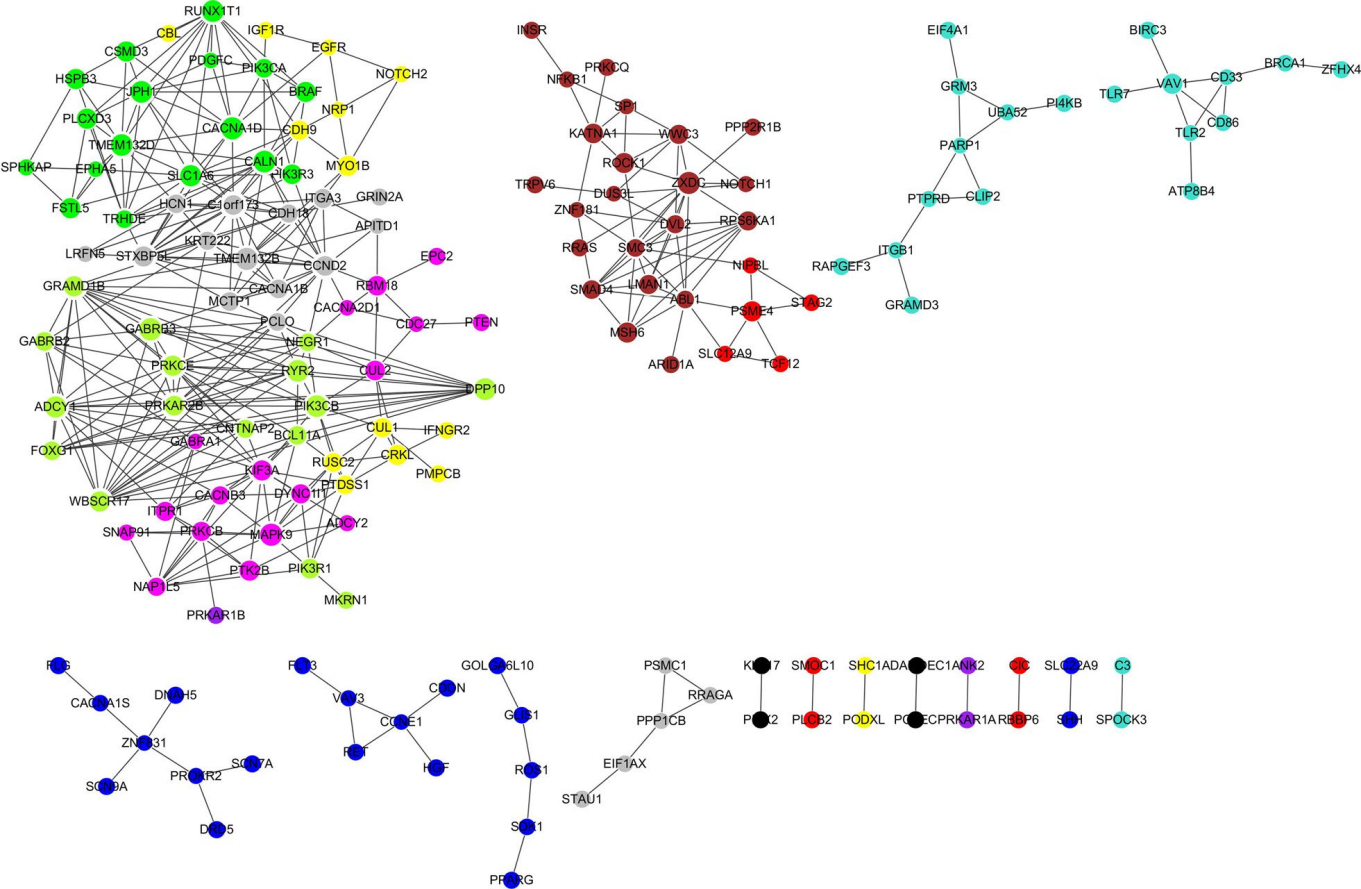
Co-expression networks of differentially expressed driver genes in normal brain tissue

In order to analyze the driver gene expression profile in glioma, RNA-seq data of 75 glioma and 17 normal brain tissues were obtained from Gill’s study. Overall, we found 428 differentially expressed driver genes, including 330 up-regulated and 98 down-regulated genes (Additional file 1: Figure S2). Next, we built co-expression networks based on the expression correlation of differentially expressed driver genes in cancer tissues and normal brain tissues respectively. The genes which have high degrees possess intensive interactions with other genes and may act as key genes in the co-expression network. As shown Fig. 3, *FSTL5, HCN1, TMEM132D, TRHDE, KRT222, CACNA1B, CDH9, SLC22A9, GABRA1* and *GABRB2* showed the strongest expression correlation with other genes in glioma tissues (Additional file 1: Table S7), while, *PRKAR2B, CCND2, C1orf173, WBSCR17, STXBP5L, PRKCE, KIF3A, GRAMD1B, SLC1A6* and *ADCY1* were the hub genes in the co-expression network of normal brain tissues (Fig. 4; Additional file 1: Table S8).

### Protein–protein interaction network analysis

In addition to co-expression analysis on driver genes at the mRNA level, we also wanted to know the interactions of driver genes in glioma at the protein level. To this aim, we applied STRING to construct a protein–protein interaction network using 685 driver genes. A high degree protein regulates or is regulated by many other proteins, suggesting an important role in the network of interactions. *SRC, ST6GAL2, PIK3CA, PIK3R1, CREBBP, TP53, SOS1, EGFR, EGR1* and *EIF1AX* are at the core of the protein–protein interaction network (Total STRING score > 29.20, Additional file 1: Table S9, Figure S3). They are responsible for regulation of programmed cell death, protein metabolic process, apoptotic process, EGFR signaling pathway and signaling transduction, suggesting they may play key roles in glioma [24].

### Copy number variations in glioma

We also obtained focal CNVs of 52 glioma samples at broad institute. Significant focal gains and deletions (q < 0.25) were found in 29 samples (29/52, 55.77%) at 5 loci (3 amplifications and 2 deletions). Among them, deletions at 9p21.3, 7p11.2 and amplification at 1q32.1 were the most frequent CNVs in glioma, with occurrence rates of 32.69% (17/52), 26.92% (14/52) and 13.46% (7/52) respectively (Additional file 1: Figure S4). 10 driver genes were involved in CNVs, including known tumor suppressors and oncogenes, such as *EGFR* (amplification, 7p11.2) and *MET* (amplification, 12q14.1). Many driver genes were also found to be implicated in the CNVs, including *PIP4K2C* (amplification, 12q14.1), *REN* (amplification, 1q32.1), *PIK3C2B* (amplification, 1q32.1), *CDKN2B* and *CDKN2A* (deletion, 9p21.3), *COL6A3* and *NEU2* (deletion, 2q37.3) and *HDAC4* (deletion, 2q37.3).

### Survival analyses in glioma

**Fig. 5:**
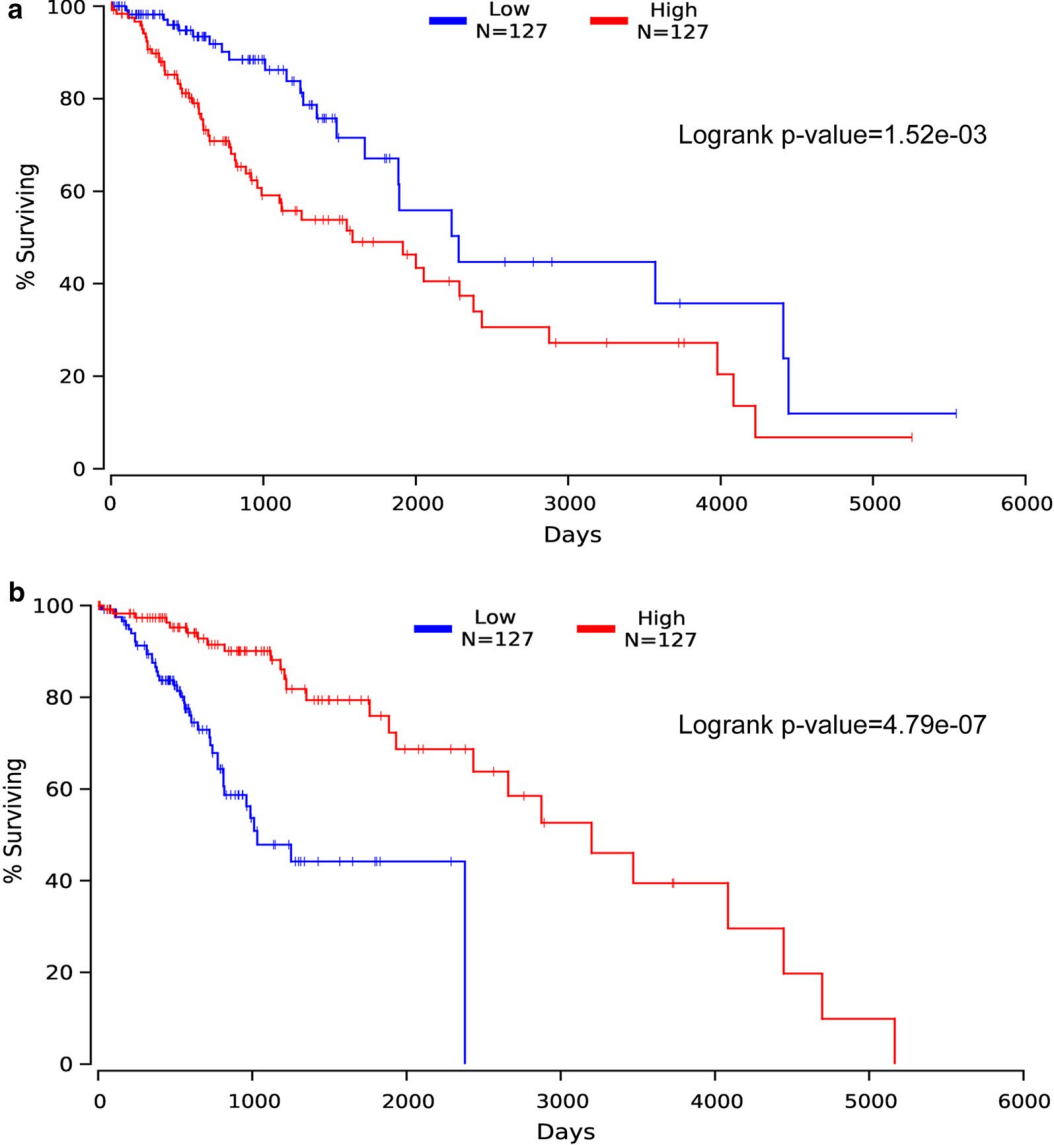
Correlation of *MSH6* and *RUNX1T1* expression with patients’ prognosis in glioma. **a** Patients with high expression of *MSH6* (log2 normalized count was above 146.48, red) showed a poor survival rate as compared to glioma patients with low expression of *MSH6* (log2 normalized count was smaller than 47.69, blue). **b** Glioma patients with high expression of *RUNX1T1* (log2 normalized count was above 1035.7, red) showed a better prognosis than patients with low expression of *RUNX1TX* (log2 normalized count was less than 759.39, blue)

We acquired RNAseq and clinical outcome data from TCGA to evaluate whether the expression of driver genes is associated to survival of glioma patients. Overall, Kaplan–Meier survival analyses showed that the expression of 268 driver genes was significantly related to clinical outcomes of glioma patients. The high expression of 162 driver genes was negatively correlated with survival rates of glioma patients, such as *SAMD9L, SAMD9, VAV3, FLNA, KDELC2, BRCA2, MAP3K1, BRCA1, LAMA2* and *PDGFD* (Additional file 1: Table S10). By contrast, 106 driver genes showed positive correlation with patients’ prognosis, such as *BMP2, CSMD3, SMOC1, FAM110B, SLC1A6, GABRB3, BAG1, SNAP91, CALN1* and *MAPK9* (Additional file 1: Table S10). 133 driver genes were up-regulated and associated to poor prognoses in glioma patients, such as *NOTCH2, STAT3, IDH1, ARID1A* and *MSH6* (Fig. 5a). On the contrary, 43 driver genes were down-regulated expression and related to favorable clinical outcomes in glioma patients, such as *PIK3R1, FLT3, PIK3R1* and *RUNX1T1* (Fig. 5b). These driver genes might be potential prognostic biomarkers for glioma patients in the future.

## Discussion

In this study, we applied OncodriveCLUST, OncodriveFM, Icages, Drgap and Dendrixto 576 pairs of glioma tumor/normal samples and identified 685 driver genes and 215 driver pathways in glioma. Only a small number of driver genes are recurrently mutated in glioma samples, such as *IDH1, TP53, ATRX, TTN, PTEN, EGFR* and *MUC16*. By comparing the list of driver genes to annotated oncogene [25] and tumor suppressor gene [26] databases, we found 76 known oncogenes, such as *BRAF, FOXO1, KRAS,MET* and *MTOR* as well as 61 tumor suppressor genes, such as *ATM, BRCA1,CHEK1, FOXO1* and *NOTCH1*. The majority of driver genes showed low or middle mutation frequencies and were first determined as driver genes in glioma, such as *BCOR, FRG1B, GABRA6* and *LRP1B*. In addition, multiple *IDH1*-dependent driver genes were also detected by Oncodrive-FM and drgap, for instance *NOTCH1* and *ARID1A*, suggesting these drivers might be therapeutic targets for *IDH1*-mutated gliomas.

In addition, we also uncovered 428 differentially expressed driver genes, including 330 up-regulated and 98 down-regulated genes, as well as 10 driver genes involved in CNVs, suggesting these genes might contribute to gliomagenesis in a variety of fashions. On the basis of the 428 differentially expressed driver genes, we built gene co-expression networks in glioma tissues and normal brain tissues. No significant loss of connections between genes were observed in glioma, which contrasts with Hao Li’s study [27], in which most of the gene–gene interactions and linkages in normal tissues had been broken or lost in the gastric cancer tissues. The inconsistent findings might be attributed to two factors. First of all, cancer types are greatly different in the two studies. Secondly, the co-expression networks were built using two distinct subset of genes, including differentially expressed driver genes in our study and differentially expressed genes in Hao’s study. The PPI network analysis indicated the top ranking genes are responsible for regulation of programmed cell death, protein metabolic process, apoptotic process, *EGFR* signaling pathway and signaling transduction, suggesting they may play pivotal roles in glioma [24].

Lastly, 268 driver genes were significantly correlated to clinical outcomes of glioma patients. Of them, two driver genes, *MSH6* and *RUNX1T1*, drew our attention, as they have been repeatedly reported to be involved in multiple cancer types [28–39]. The *MSH6* is a member of mismatch repair (*MMR*) genes, germline mutations in *MMR* genes, predominantly in *MLH1, MSH2* and *MSH6*, are responsible for hereditary nonpolyposis colorectal cancer [28, 29], prostate cancer [30] and endometrial cancer [31]. In line with our study, high expression of *MSH6* was significantly associated with poor survival rates in melanoma [32] and osteosarcoma [33]. The second gene, *RUNX1T1*, encodes a member of myeloid translocation genes. The chromosomal translocation t(8;21)(q22;q22) generates the *RUNX1/RUNX1T1* fusion gene, which supports human haematopoietic stem cell self-renewal as well as leukaemic proliferation and clonogenicity in vivo [34–37]. The expression of *RUNX1T1* was severely down-regulated in colorectal cancer (CRC), increased expression of *RUNX1T1* suppressed cellular proliferation and sensitized CRC cells to 5-fluorouracil [38]. *RUNX1T1* was frequently hypermethylated in ovarian tumors with high clinical stages and primary ovarian cancer-initiating cells. Enhanced *RUNX1T1* expression inhibited ovarian cancer cell growth [39]. The results obtained in our study in combination with published reports support that *MSH6* and *RUNX1T1* have oncogenic and tumor suppressive functions respectively in cancers.

## Conclusions

In conclusion, we performed an integrative study on the set of driver genes detected by five distinct computational tools, which enhanced our understanding of tumorigenesis and progression of glioma. The driver genes and pathways identified herein such as *MSH6* and *RUNX1T1* might be candidate prognostic biomarkers and therapeutic targets in glioma.

## Additional file

**Additional file 1: Figure S1.** Driver genes in IDH1-mutated, non-IDH1-mutated and total glioma groups. A. The overlap of driver genes predicted by OncodriveCLUST in the three groups; B. The overlap of driver genes predicted by OncodriveFM in the three groups; C. The overlap of driver genes predicted by Icages in the three groups; D. The overlap of driver genes predicted by Drgap in the three groups; E. The overlap of driver genes predicted by Dendrix in the three groups; F. The overlap of driver pathways predicted by Dendrix in the three groups. **Figure S2.** Expression profiling of 428 driver genes between glioma and normal brain samples, expression values of each gene was presented as log(FPKM + 0.001). **Figure S3.** The protein–protein interaction network of driver genes. Network nodes represent proteins, network edges represent protein–protein associations. Notably, disconnected nodes were disabled in the network. Colored nodes refer to query proteins and first shell of interactors, white nodes are second shell of interactors. Blue lines represent known interactions from curated Kyoto Encyclopedia of Genes and Genomes database. Pink lines represent experimentally determined protein–protein interactions. Green lines denote genes that are frequently observed in each other’s genomic neighborhood. Black lines stand for genes whose expression is correlated across a large number of experiments. Dark blue gene families show similar occurrence patterns across genomes, light blue proteins show amino acid sequence similarity. **Figure S4.** The frequencies of copy number variations in 52 glioma samples.

## Abbreviations

DEGs: differentially expressed genes
GO: gene ontology
PPI: protein–protein interaction
CNV: copy number variation
FPKM: fragments per kilobase of exon per million fragments mapped
FDR: false discovery rate
CRC: colorectal cancer
